# 2-Chloro-1-[4-(2,4-difluoro­benz­yl)piperazin-1-yl]ethanone

**DOI:** 10.1107/S1600536811052597

**Published:** 2011-12-10

**Authors:** Bo Zhang, Guri L. V. Damu, Jing-Song Lv, Cheng-He Zhou

**Affiliations:** a Laboratory of Bioorganic & Medicinal Chemistry, School of Chemistry and Chemical Engineering, Southwest University, Chongqing 400715, People’s Republic of China

## Abstract

In the title mol­ecule, C_13_H_15_ClF_2_N_2_O, the piperazine ring is in a chair conformation with the 2,4-difluoro­benzyl and chloro­acetyl substituents in equatorial positions.

## Related literature

For the synthesis, see: Gan *et al.* (2010 ▶). For applications of piperazine derivatives, see: Gan, Cai & Zhou (2009 ▶); Cai *et al.* (2009 ▶); Gan, Lu, & Zhou (2009 ▶).
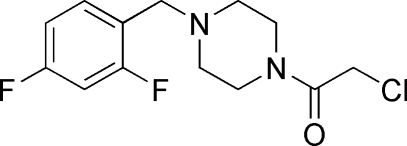


## Experimental

### 

#### Crystal data

C_13_H_15_ClF_2_N_2_O
*M* *_r_* = 288.72
Orthorhombic, 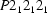

*a* = 7.895 (2) Å
*b* = 8.512 (2) Å
*c* = 19.884 (5) Å
*V* = 1336.2 (6) Å^3^
*Z* = 4
Mo *K*α radiation
μ = 0.30 mm^−1^
*T* = 296 K
0.30 × 0.25 × 0.24 mm

#### Data collection

Bruker SMART CCD diffractometer
Absorption correction: multi-scan (*SADABS*; Sheldrick, 1996 ▶) *T*_min_ = 0.915, *T*_max_ = 0.931
5743 measured reflections
2324 independent reflections
2238 reflections with *I* > 2σ(*I*)
*R*_int_ = 0.026

#### Refinement

*R*[*F* ^2^ > 2σ(*F* ^2^)] = 0.031
*wR*(*F* ^2^) = 0.086
*S* = 1.07
2324 reflections
220 parameters
H atoms treated by a mixture of independent and constrained refinement
Δρ_max_ = 0.37 e Å^−3^
Δρ_min_ = −0.18 e Å^−3^
Absolute structure: Flack (1983 ▶), 938 Friedel pairs
Flack parameter: 0.05 (7)

### 

Data collection: *SMART* (Bruker, 2001 ▶); cell refinement: *SAINT* (Bruker, 2001 ▶); data reduction: *SAINT*; program(s) used to solve structure: *SHELXS97* (Sheldrick, 2008 ▶); program(s) used to refine structure: *SHELXL97* (Sheldrick, 2008 ▶); molecular graphics: *PLATON* (Spek, 2009 ▶); software used to prepare material for publication: *SHELXTL* (Sheldrick, 2008 ▶).

## Supplementary Material

Crystal structure: contains datablock(s) global, I. DOI: 10.1107/S1600536811052597/lh5389sup1.cif

Structure factors: contains datablock(s) I. DOI: 10.1107/S1600536811052597/lh5389Isup2.hkl

Supplementary material file. DOI: 10.1107/S1600536811052597/lh5389Isup3.cml

Additional supplementary materials:  crystallographic information; 3D view; checkCIF report

## Figures and Tables

**Fig. 1.**
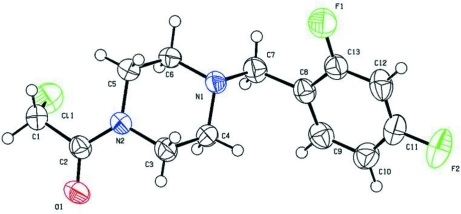
The molecular structure of the title compound showing 50% ellipsoids.

## supplementary crystallographic information

### Comment

The piperazine ring is present in many clinical drugs (Cai *et al.*, 2009). The incorporation of a piperazine moiety gernerally improves physicochemical properties, and thereby enhances biological activities (Gan, Cai & Zhou, 2009). The piperazine moiety is extensively employed to construct various bioactive molecules (Gan, Lu & Zhou, 2009). Our interest is to develop azole-containing piperazine derivatives as medicinal agents (Gan *et al.*, 2010). Herein we report the crystal structure of the title compound (I).

In the molecular structure (Fig. 1) of (I) the piperazine ring is in a chair conformation, in which the 2,4-difluorobenzyl and chloroacetyl groups are located in equatorial positions.

### Experimental

Compound (I) was synthesized according to the procedure of Gan *et al.* (2010). Single crystals suitable for X-ray diffraction analysis were grown in a mixed solution petroleum ether and ethyl acetate by slow evaporation at room temperature.

### Refinement

The hydrogen atoms attached to the benzene ring were placed in calculated positions with C—H = 0.93 Å and U_iso_(H) = 1.2U_eq_(C). All other H atoms were refined independently with isotropic displacement parameters.

### Crystal data

**Table:**

C_13_H_15_ClF_2_N_2_O	*F*(000) = 600
*M**_r_* = 288.72	*D*_x_ = 1.435 Mg m^−^^3^
Orthorhombic, *P*2_1_2_1_2_1_	Mo *K*α radiation, λ = 0.71073 Å
Hall symbol: P 2ac 2ab	Cell parameters from 4078 reflections
*a* = 7.895 (2) Å	θ = 3.2–27.4°
*b* = 8.512 (2) Å	µ = 0.30 mm^−^^1^
*c* = 19.884 (5) Å	*T* = 296 K
*V* = 1336.2 (6) Å^3^	Block, colorless
*Z* = 4	0.30 × 0.25 × 0.24 mm

### Data collection

**Table:**

Bruker SMART CCD diffractometer	2324 independent reflections
Radiation source: fine-focus sealed tube	2238 reflections with *I* > 2σ(*I*)
graphite	*R*_int_ = 0.026
φ and ω scans	θ_max_ = 25.0°, θ_min_ = 2.1°
Absorption correction: multi-scan (*SADABS*; Sheldrick, 1996)	*h* = −9→8
*T*_min_ = 0.915, *T*_max_ = 0.931	*k* = −8→10
5743 measured reflections	*l* = −21→23

### Refinement

**Table:**

Refinement on *F*^2^	Secondary atom site location: difference Fourier map
Least-squares matrix: full	Hydrogen site location: inferred from neighbouring sites
*R*[*F*^2^ > 2σ(*F*^2^)] = 0.031	H atoms treated by a mixture of independent and constrained refinement
*wR*(*F*^2^) = 0.086	*w* = 1/[σ^2^(*F*_o_^2^) + (0.0501*P*)^2^ + 0.1826*P*] where *P* = (*F*_o_^2^ + 2*F*_c_^2^)/3
*S* = 1.07	(Δ/σ)_max_ < 0.001
2324 reflections	Δρ_max_ = 0.37 e Å^−^^3^
220 parameters	Δρ_min_ = −0.18 e Å^−^^3^
0 restraints	Absolute structure: Flack (1983), 938 Friedel pairs
Primary atom site location: structure-invariant direct methods	Flack parameter: 0.05 (7)

### Special details

**Table:**

Geometry. All e.s.d.'s (except the e.s.d. in the dihedral angle between two l.s. planes) are estimated using the full covariance matrix. The cell e.s.d.'s are taken into account individually in the estimation of e.s.d.'s in distances, angles and torsion angles; correlations between e.s.d.'s in cell parameters are only used when they are defined by crystal symmetry. An approximate (isotropic) treatment of cell e.s.d.'s is used for estimating e.s.d.'s involving l.s. planes.
Refinement. Refinement of *F*^2^ against ALL reflections. The weighted *R*-factor *wR* and goodness of fit *S* are based on *F*^2^, conventional *R*-factors *R* are based on *F*, with *F* set to zero for negative *F*^2^. The threshold expression of *F*^2^ > σ(*F*^2^) is used only for calculating *R*-factors(gt) *etc*. and is not relevant to the choice of reflections for refinement. *R*-factors based on *F*^2^ are statistically about twice as large as those based on *F*, and *R*- factors based on ALL data will be even larger.

### Fractional atomic coordinates and isotropic or equivalent isotropic displacement parameters (Å^2^)

**Table:**

	*x*	*y*	*z*	*U*_iso_*/*U*_eq_	
Cl1	0.26806 (8)	1.41412 (7)	0.14714 (3)	0.06679 (19)	
N1	0.53726 (18)	0.88254 (16)	0.14628 (8)	0.0392 (3)	
F1	0.86597 (19)	0.62487 (19)	0.15098 (7)	0.0786 (4)	
N2	0.25240 (19)	1.04611 (18)	0.20166 (8)	0.0441 (4)	
C7	0.6577 (3)	0.8543 (2)	0.09087 (11)	0.0466 (4)	
C2	0.1248 (2)	1.1492 (2)	0.19486 (9)	0.0397 (4)	
C4	0.3630 (2)	0.8503 (2)	0.12429 (10)	0.0437 (4)	
C6	0.5486 (2)	1.0480 (2)	0.16685 (11)	0.0458 (4)	
O1	−0.01636 (16)	1.11102 (17)	0.17464 (8)	0.0559 (4)	
C3	0.2357 (3)	0.8832 (2)	0.17911 (11)	0.0482 (4)	
F2	0.6783 (2)	0.21756 (16)	0.01314 (7)	0.0834 (5)	
C1	0.1573 (3)	1.3191 (2)	0.21356 (10)	0.0474 (4)	
C13	0.7660 (2)	0.5758 (2)	0.10072 (9)	0.0477 (4)	
C9	0.5670 (3)	0.6280 (2)	0.01646 (10)	0.0504 (5)	
H9	0.4984	0.6980	−0.0070	0.061*	
C5	0.4260 (2)	1.0821 (3)	0.22316 (11)	0.0509 (5)	
C8	0.6638 (2)	0.6844 (2)	0.06941 (8)	0.0416 (4)	
C10	0.5682 (3)	0.4712 (3)	−0.00294 (10)	0.0539 (5)	
H10	0.5005	0.4354	−0.0380	0.065*	
C11	0.6726 (3)	0.3717 (2)	0.03146 (10)	0.0540 (5)	
C12	0.7734 (3)	0.4185 (2)	0.08374 (10)	0.0576 (5)	
H12	0.8430	0.3483	0.1066	0.069*	
H2M	0.219 (3)	1.327 (3)	0.2523 (10)	0.049 (6)*	
H1M	0.049 (3)	1.374 (3)	0.2171 (10)	0.048 (5)*	
H9M	0.451 (3)	1.011 (3)	0.2616 (12)	0.056 (6)*	
H5M	0.328 (2)	0.920 (2)	0.0833 (9)	0.038 (5)*	
H4M	0.265 (3)	0.814 (3)	0.2201 (11)	0.064 (6)*	
H10M	0.432 (3)	1.185 (3)	0.2347 (12)	0.064 (7)*	
H7M	0.665 (3)	1.071 (2)	0.1839 (9)	0.045 (5)*	
H6M	0.362 (3)	0.745 (3)	0.1093 (11)	0.062 (7)*	
H8M	0.524 (3)	1.123 (2)	0.1293 (10)	0.048 (5)*	
H3M	0.134 (3)	0.868 (3)	0.1630 (11)	0.057 (6)*	
H11M	0.761 (3)	0.886 (3)	0.1060 (10)	0.051 (5)*	
H12M	0.619 (3)	0.919 (3)	0.0531 (13)	0.061 (6)*	

### Atomic displacement parameters (Å^2^)

**Table:**

	*U*^11^	*U*^22^	*U*^33^	*U*^12^	*U*^13^	*U*^23^
Cl1	0.0769 (4)	0.0526 (3)	0.0708 (3)	−0.0112 (3)	0.0027 (3)	0.0112 (2)
N1	0.0336 (7)	0.0325 (7)	0.0514 (8)	0.0009 (6)	0.0024 (6)	−0.0012 (6)
F1	0.0775 (9)	0.0846 (10)	0.0737 (8)	0.0243 (8)	−0.0276 (7)	−0.0206 (8)
N2	0.0328 (7)	0.0415 (8)	0.0581 (8)	0.0027 (7)	−0.0002 (7)	−0.0081 (7)
C7	0.0422 (10)	0.0400 (10)	0.0577 (11)	−0.0014 (8)	0.0096 (9)	0.0031 (9)
C2	0.0351 (8)	0.0415 (9)	0.0424 (8)	0.0014 (8)	0.0052 (7)	0.0008 (7)
C4	0.0375 (9)	0.0323 (9)	0.0611 (11)	−0.0029 (8)	−0.0004 (8)	−0.0060 (8)
C6	0.0321 (9)	0.0385 (10)	0.0667 (12)	−0.0006 (7)	−0.0065 (8)	−0.0067 (9)
O1	0.0328 (6)	0.0533 (8)	0.0815 (9)	0.0013 (6)	−0.0034 (6)	−0.0032 (7)
C3	0.0361 (9)	0.0376 (9)	0.0710 (12)	−0.0001 (8)	0.0047 (9)	−0.0024 (9)
F2	0.1251 (14)	0.0440 (7)	0.0812 (9)	0.0043 (8)	0.0120 (9)	−0.0143 (7)
C1	0.0461 (10)	0.0431 (10)	0.0532 (11)	0.0057 (9)	0.0024 (9)	−0.0072 (9)
C13	0.0456 (9)	0.0524 (11)	0.0451 (9)	0.0064 (10)	−0.0002 (8)	−0.0044 (8)
C9	0.0508 (10)	0.0531 (12)	0.0474 (10)	0.0074 (9)	−0.0017 (8)	0.0041 (9)
C5	0.0394 (10)	0.0492 (12)	0.0643 (12)	0.0064 (9)	−0.0108 (9)	−0.0151 (10)
C8	0.0386 (9)	0.0418 (10)	0.0444 (9)	0.0017 (8)	0.0096 (7)	0.0030 (8)
C10	0.0562 (12)	0.0567 (12)	0.0487 (10)	−0.0027 (10)	0.0024 (9)	−0.0083 (9)
C11	0.0689 (13)	0.0396 (10)	0.0535 (10)	−0.0009 (10)	0.0147 (9)	−0.0055 (9)
C12	0.0688 (13)	0.0498 (11)	0.0542 (11)	0.0208 (11)	0.0009 (10)	0.0031 (9)

### Geometric parameters (Å, °)

**Table:**

Cl1—C1	1.778 (2)	C6—H8M	1.00 (2)
N1—C4	1.469 (2)	C3—H4M	1.03 (2)
N1—C6	1.469 (2)	C3—H3M	0.88 (3)
N1—C7	1.475 (2)	F2—C11	1.362 (2)
F1—C13	1.340 (2)	C1—H2M	0.91 (2)
N2—C2	1.343 (2)	C1—H1M	0.98 (2)
N2—C3	1.463 (2)	C13—C8	1.376 (3)
N2—C5	1.468 (2)	C13—C12	1.382 (3)
C7—C8	1.509 (3)	C9—C8	1.387 (3)
C7—H11M	0.91 (2)	C9—C10	1.389 (3)
C7—H12M	0.98 (3)	C9—H9	0.9300
C2—O1	1.228 (2)	C5—H9M	1.00 (2)
C2—C1	1.516 (3)	C5—H10M	0.90 (3)
C4—C3	1.509 (3)	C10—C11	1.365 (3)
C4—H5M	1.046 (19)	C10—H10	0.9300
C4—H6M	0.94 (3)	C11—C12	1.368 (3)
C6—C5	1.508 (3)	C12—H12	0.9300
C6—H7M	1.00 (2)		
			
C4—N1—C6	108.60 (14)	H4M—C3—H3M	114 (2)
C4—N1—C7	110.54 (15)	C2—C1—Cl1	109.57 (13)
C6—N1—C7	108.94 (14)	C2—C1—H2M	111.4 (14)
C2—N2—C3	121.37 (16)	Cl1—C1—H2M	109.4 (13)
C2—N2—C5	126.42 (16)	C2—C1—H1M	108.8 (13)
C3—N2—C5	111.78 (15)	Cl1—C1—H1M	105.6 (12)
N1—C7—C8	112.82 (15)	H2M—C1—H1M	111.9 (18)
N1—C7—H11M	106.6 (13)	F1—C13—C8	118.23 (17)
C8—C7—H11M	110.3 (14)	F1—C13—C12	117.35 (17)
N1—C7—H12M	106.2 (14)	C8—C13—C12	124.42 (18)
C8—C7—H12M	109.6 (14)	C8—C9—C10	122.64 (19)
H11M—C7—H12M	111.4 (19)	C8—C9—H9	118.7
O1—C2—N2	122.76 (17)	C10—C9—H9	118.7
O1—C2—C1	119.10 (16)	N2—C5—C6	110.06 (16)
N2—C2—C1	118.14 (16)	N2—C5—H9M	106.3 (14)
N1—C4—C3	111.97 (16)	C6—C5—H9M	109.0 (13)
N1—C4—H5M	112.1 (10)	N2—C5—H10M	108.9 (16)
C3—C4—H5M	106.2 (10)	C6—C5—H10M	109.9 (16)
N1—C4—H6M	106.0 (15)	H9M—C5—H10M	113 (2)
C3—C4—H6M	113.6 (14)	C13—C8—C9	115.72 (18)
H5M—C4—H6M	106.9 (17)	C13—C8—C7	122.34 (17)
N1—C6—C5	110.60 (17)	C9—C8—C7	121.95 (17)
N1—C6—H7M	109.5 (12)	C11—C10—C9	117.5 (2)
C5—C6—H7M	107.6 (11)	C11—C10—H10	121.3
N1—C6—H8M	112.9 (11)	C9—C10—H10	121.3
C5—C6—H8M	107.8 (12)	F2—C11—C10	118.9 (2)
H7M—C6—H8M	108.2 (17)	F2—C11—C12	117.6 (2)
N2—C3—C4	109.69 (15)	C10—C11—C12	123.4 (2)
N2—C3—H4M	106.1 (13)	C11—C12—C13	116.28 (19)
C4—C3—H4M	108.3 (14)	C11—C12—H12	121.9
N2—C3—H3M	109.5 (15)	C13—C12—H12	121.9
C4—C3—H3M	108.7 (15)		
			
C4—N1—C7—C8	−64.7 (2)	N1—C6—C5—N2	−58.6 (2)
C6—N1—C7—C8	176.09 (16)	F1—C13—C8—C9	178.12 (17)
C3—N2—C2—O1	6.0 (3)	C12—C13—C8—C9	−1.7 (3)
C5—N2—C2—O1	177.8 (2)	F1—C13—C8—C7	−1.6 (3)
C3—N2—C2—C1	−174.52 (18)	C12—C13—C8—C7	178.66 (19)
C5—N2—C2—C1	−2.7 (3)	C10—C9—C8—C13	1.8 (3)
C6—N1—C4—C3	−58.6 (2)	C10—C9—C8—C7	−178.50 (18)
C7—N1—C4—C3	−178.10 (15)	N1—C7—C8—C13	−85.7 (2)
C4—N1—C6—C5	59.1 (2)	N1—C7—C8—C9	94.6 (2)
C7—N1—C6—C5	179.51 (16)	C8—C9—C10—C11	−1.3 (3)
C2—N2—C3—C4	117.63 (19)	C9—C10—C11—F2	−179.20 (19)
C5—N2—C3—C4	−55.3 (2)	C9—C10—C11—C12	0.5 (3)
N1—C4—C3—N2	56.8 (2)	F2—C11—C12—C13	179.39 (19)
O1—C2—C1—Cl1	−101.22 (18)	C10—C11—C12—C13	−0.4 (3)
N2—C2—C1—Cl1	79.24 (19)	F1—C13—C12—C11	−178.81 (18)
C2—N2—C5—C6	−115.8 (2)	C8—C13—C12—C11	1.0 (3)
C3—N2—C5—C6	56.7 (2)		
